# Differential Associations of Erythrocyte Membrane Saturated Fatty Acids with Glycemic and Lipid Metabolic Markers in a Chinese Population: A Cross-Sectional Study

**DOI:** 10.3390/nu16101507

**Published:** 2024-05-16

**Authors:** Shixin Wu, Huiru Luo, Juncheng Zhong, Mengyang Su, Xiaoying Lai, Zheqing Zhang, Quan Zhou

**Affiliations:** 1 School of Public Health, Guangzhou Medical University, Guangzhou 510180, China; wushixin@stu.gzhmu.edu.cn (S.W.); luozi168@foxmail.com (H.L.);; 2 Department of Nutrition and Food Hygiene, Guangdong Provincial Key Laboratory of Tropical Disease Research, School of Public Health, Southern Medical University, Guangzhou 510515, China; sumyang1137@163.com; 3 Nanfang Hospital, Southern Medical University, Guangzhou 510515, China; 617367790@163.com

**Keywords:** erythrocyte membrane, saturated fatty acids, glycemic, lipid, metabolic markers, cross-sectional

## Abstract

Mounting evidence indicates a complex link between circulating saturated fatty acids (SFAs) and cardiovascular disease (CVD) risk factors, but research on erythrocyte membrane SFA associations with metabolic markers remains limited. Our study sought to investigate the correlations between erythrocyte membrane SFAs and key metabolic markers within glycemic and lipid metabolism in a Chinese population of 798 residents aged 41 to 71 from Guangzhou. Using gas chromatography–mass spectrometry, we assessed the erythrocyte membrane saturated fatty acid profile and performed multiple linear regression to evaluate the relationship between different SFA subtypes and metabolic markers. Our findings revealed that the odd-chain SFA group (C15:0 + C17:0) exhibited negative associations with fasting blood glucose (FBG), homeostatic model assessment for insulin resistance (HOMA-IR), and triglycerides (TG). Conversely, the very-long-chain SFA group (C20:0 + C22:0 + C23:0 + C24:0) exhibited positive associations with fasting insulins (FINS), HOMA-IR, total cholesterol (TC), and low-density lipoprotein cholesterol (LDL-C). Furthermore, there was no evidence supporting an association between the even-chain group (C14:0 + C16:0 + C18:0) and metabolic markers. Our findings suggest that different subtypes of SFAs have diverse effects on glycemic and lipid metabolic markers, with odd-chain SFAs associated with a lower metabolic risk. However, the results concerning the correlations between even-chain SFAs and very-long-chain SFAs with markers of glycemic and lipid metabolism pathways are confusing, highlighting the necessity for further exploration and investigation.

## 1. Introduction

Cardiovascular disease (CVD) poses a significant challenge to global health, comprising a range of conditions that affect the heart and blood vessels, including coronary heart disease, ischemic heart disease, pulmonary heart disease, hypertension, and stroke. Despite advances in understanding the pathophysiology and associated risk factors of CVD over the decades [1], it continues to be the leading cause of global mortality. To provide examples, in 2017, CVD claimed 18 million lives globally, and by 2019, China alone accounted for 330 million CVD cases [2]. These numbers underscore the urgent need for effective strategies to address the CVD burden.

CVD occurrence is linked to numerous risk factors, including age, smoking, alcohol consumption, hypertension, dyslipidemia, diabetes, obesity, and others [3]. However, the role of circulating saturated fatty acids (SFAs) in CVD etiology has gained increasing attention in recent years. Numerous studies have investigated the association between circulating SFAs and CVD risks, revealing inconsistent findings regarding different individual SFAs and their relationship with CVD risks [4,5,6,7,8]. For instance, a study found that circulating odd-chain SFAs were inversely related to coronary heart disease (CHD), whereas C18:0 was strongly positively associated with CHD [4]. Similarly, higher levels of circulating very-long-chain SFAs have been associated with a reduced risk of sudden cardiac arrest and heart failure [5,6]. A recent meta-analysis identified positive associations between circulating C14:0 and C16:0 and CVD, but no significant associations for other SFAs like C15:0, C16:0, C18:0, C20:0, and C22:0 were observed [8].

Despite the extensive research on the association between circulating SFAs and CVD risks, there has been limited exploration into the correlations between erythrocyte membrane SFAs and specific cardiovascular risk factors, particularly among Asian populations. Moreover, since the pathogenesis of CVD involves multiple metabolic pathways and intermediary markers, including glycemic and lipid metabolic markers [9], there is a critical need for a comprehensive examination of erythrocyte membrane SFAs and their associations with metabolic markers.

Thus, we conducted a cross-sectional study involving permanent residents of Guangzhou, China. Our research sought to investigate the associations of individual erythrocyte membrane SFAs and corresponding SFA groups with several metabolic markers related to glycemic and lipid metabolism. By elucidating these associations, our study seeks to contribute to a better comprehension of the role of SFAs in the pathogenesis of CVD, particularly in relation to glycemic and lipid metabolism pathways.

## 2. Materials and Methods

### 2.1. Study Design and Population

This study was a community-based cross-sectional study aimed at assessing the nutritional and health status of middle-aged and elderly individuals in Guangzhou, China. Participants were actively recruited by the Southern Hospital Health Management Center from November 2018 to August 2019. Eligible participants were residents of urban Guangzhou for over 2 years and aged between 41 and 71 years. Moreover, within the past 5 years, none of the participants had been diagnosed with cardiovascular or metabolic diseases, respiratory diseases, chronic kidney diseases, or cancer based on records from hospitals or community health centers. The exclusion criteria were as follows: (1) A self-reported history and confirmed history of cardiovascular disease (including coronary heart disease, myocardial infarction, angina pectoris, ischemic heart disease), stroke, diabetes, and cancers. (2) The absence of available fatty acid data and covariate data. Initially, 875 subjects were recruited, and 77 individuals were subsequently excluded based on predetermined exclusion criteria. Thus, a total of 798 participants were included in this study. Written informed consent was obtained from all study participants, and the study protocol received approval from the Institutional Research Board of the hospital.

### 2.2. Data Collection

We employed lifestyle questionnaires to evaluate demographics, smoking status, alcohol drinking, medical history, and family history of diseases (including hypertension, type 2 diabetes, dyslipidemia, and coronary heart disease). Smokers were defined as individuals who had smoked at least one cigarette per day for a minimum of six months. Alcohol drinkers consumed alcohol at least once a week continuously for six months. The participants’ physical activity levels were assessed using a 19-item physical activity questionnaire, measuring metabolic equivalent hours per day (MET·h/d), which included exercise, leisure-time activities, housework, occupation-related activities, and other daily activities [10]. Physical activity was classified as light, moderate, and vigorous according to the tertiles of the metabolic equivalent of the task (MET). A 79-item food frequency questionnaire (FFQ) [11] was used to evaluate the participants’ usual dietary intake over the past year during the interview for controls. Participants were requested to report the frequency and intake quantity of each food consumed, delineated in terms of per day, per week, per month, or per year. Visual aids, including food photographs and portion sizes, were offered to aid participants in quantifying their consumption. These values were then converted to represent the daily intake. The total energy intake and dietary intake were determined based on the Chinese Food Composition Table of 2009 [12]. Weight was measured using a digital scale in kilograms, with participants wearing light clothing and no shoes, while height was measured in meters using a stadiometer. Body mass index (BMI) was calculated by dividing weight in kilograms by height in meters squared (kg/m^2^). The waist circumference was measured at the midpoint between the iliac crest and the costal margin with the participant in a standing position. Central obesity was defined as a waist circumference of 90 cm or more for men and 85 cm or more for women.

### 2.3. Laboratory Measurements

Blood samples collected after a 12-h overnight fast were preserved in vacuum tubes containing ethylenediaminetetraacetic acid. Within a two-hour window post-collection, red blood cells (RBCs) were isolated via centrifugation at 1500× *g* for 15 min at 4 °C and subsequently stored at −80 °C until further analysis. For the assessment of fatty acid composition in the erythrocyte membrane, lipids were extracted from the stored RBCs and converted into fatty acid methyl esters (FAME), as previously reported [11,13]. The RBCs underwent thawing and were subjected to hemolysis in a hypotonic Tris-HCl buffer with a concentration of 10 mmol/L and a pH of 7.4. This process was conducted at 4 °C for a duration of 2 h. For post-hemolysis, a membrane pellet was obtained through ultracentrifugation at 12,000 rpm for 30 min at 4 °C. Lipids were then extracted from the membrane pellet using a chloroform and methanol mixture in a ratio of 2:1 (*v*/*v*), supplemented with 0.005% butylated hydroxytoluene. The resulting extract was subsequently dried using nitrogen gas. Fatty acid methyl esters were derived by incubating the dried extract with a 14% boron trifluoride ether/methanol solution in a ratio of 1:3 (*v*/*v*) at a temperature of 100 °C for 5 min. The FAMEs were then extracted into 1 mL of hexane, followed by solvent evaporation to dryness. Finally, the FAMEs were redissolved in hexane for gas chromatography-mass spectrometry analysis (GC-MS 2010SE, DB-23 capillary column: 60 m length, 0.25 mm internal diameter, and 0.15 mm film thickness; Agilent, Santa Clara, CA, USA) employing commercial standards (Nu-Chek Prep, Elysian, MN, USA) to ascertain erythrocyte membrane FA relative concentrations (proportionate values). The relative concentrations of individual FAs were computed as a percentage of total FAs, including odd-chain SFAs, even-chain SFAs, and very-long-chain SFAs, which were determined as the sum of the concentrations of individual SFAs of the corresponding type.

Serum triglycerides (TG), total cholesterol (TC), high-density lipoprotein cholesterol (HDL-C), low-density lipoprotein cholesterol (LDL-C), and fasting blood glucose (FBG) levels were analyzed using colorimetric methods with a Roche Cobas 8000 c702 automated analyzer (Roche Diagnostics GmbH, Shanghai, China). Serum insulin was measured through an immunoenzymometric assay using the Monobind kit (Monobind, Lake Forest, CA, USA). Insulin resistance was assessed by calculating the homeostatic model assessment for insulin resistance (HOMA-IR) using the following formula:

HOMA-IR = (fasting insulin (μU/mL) × fasting blood glucose (mmol/L))/22.5

### 2.4. Statistical Analysis

We analyzed nine specific SFAs (C14:0, C15:0, C16:0, C17:0, C18:0, C20:0, C22:0, C23:0, and C24:0) and added three additional exposures based on groupings of odd-chain SFA (sum of C15:0 and C17:0), even-chain SFA (sum of C14:0, C16:0, and C18:0), and very-long-chain SFA (sum of C20:0, C22:0, C23:0, and C24:0). The categorizations of the SFAs were informed by an analysis of EPIC-InterAct [14]. We investigated the disparity in the distribution of serum metabolic biomarkers and erythrocyte membrane saturated fatty acid groups across different categories of sex, age, and BMI using either the *t*-test or Mann–Whitney test for continuous variables. Spearman’s rank correlation coefficients were calculated between individual erythrocyte membrane SFAs.

We employed linear regression analysis to evaluate the relationships between each erythrocyte membrane SFA and the seven metabolic markers, including three glycemic markers (FBG, FINS, and HOMA-IR) and four lipid markers (TG, TC, HDL-C, and LDL-C). Associations were quantified as the difference in each metabolic marker (measured in standard deviation (SD) units) per 1 SD difference in each fatty acid. Variables (FBG, FINS, HOMA-IR, TG) displaying skewed distributions were log-transformed prior to analysis. Model 1: unadjusted; Model 2: adjusted for age, sex, and BMI; Model 3: Model 2 plus physical activity, smoking status, alcohol drinking, family history of diseases, and total energy intake.

We also examined the potential interaction between the three SFA groups (odd-chain, even-chain, and very-long-chain SFAs) and age, sex, alcohol drinking, and physical activity. Interactions were tested by adding interaction terms in Model 3, and we conducted the stratified analyses when a significant interaction (*p* < 0.05) was observed.

We also performed the following various sensitivity analyses based on Model 3: (3a) we included additional dietary variables (dairy intake, red and processed meat, fruit and vegetable intake, and vegetable oil intake); (3b) we adjusted for dietary fiber intake as a covariate; (3c) we considered hyperlipidemia status as a covariate; (3d) we examined the influence of central obesity; (3e) we replaced BMI with central obesity as a marker of adiposity; and (3f) we conducted mutual adjustments for the other SFA groups.

All analyses were performed using IBM SPSS software version 25.0 (SPSS, IBM, New York, NY, USA). Statistical significance was defined as a two-tailed *p*-value < 0.05.

## 3. Results

### 3.1. Population Characteristics

Table 1 presents the baseline characteristics of the study participants. The participants had an average age of 55.12 ± 7.75 years, with a gender distribution of 68.5% females and 31.5% males in the study population. Women exhibited more favorable metabolism profiles compared to men, with higher levels of HDL-C and lower levels of TG. No variations in metabolic markers were observed across different age groups. In contrast, significant differences in metabolic markers were noted between various BMI groups, except for TC and LDL-C. Furthermore, significant differences were noted in odd-chain SFAs concerning sex and BMI. Specifically, odd-chain SFA concentrations were lower in males and in individuals with a higher BMI. The even-chain SFA group and C23:0 displayed variations across different age groups, with concentrations being lower in the older age group (Table 2). The very-long-chain SFA group was positively correlated with the odd-chain SFA group while being negatively correlated with the even-chain SFA group (Table S1).

### 3.2. Association of Erythrocyte Membrane SFAs with Metabolic Markers

#### 3.2.1. Odd-Chain SFAs

**Figure 1:**
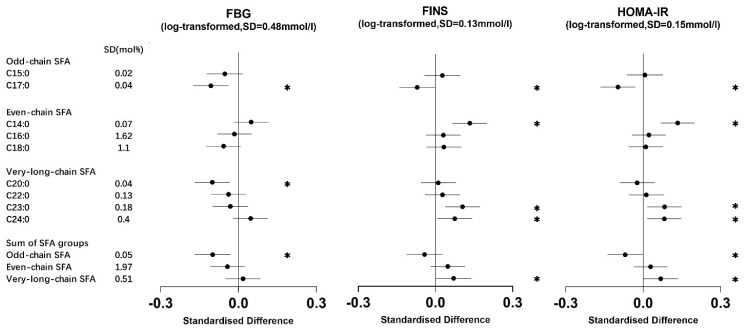
Associations of erythrocyte membrane saturated fatty acids (SFAs) with fasting glucose, fasting insulin, and insulin resistance. The standardized difference represents the change (in standard deviation units of the metabolic marker) per one standard deviation change in each SFA. The error bars depict 95% confidence intervals (CI). * signifies that the association had a *p*-value of <0.05. Models were adjusted for age, sex, BMI, smoking status, alcohol drinking, physical activity, family history of diseases, and total energy intake; FBG, FINS, and HOMA-IR were log-transformed. FBG: fasting blood glucose; FINS: fasting insulins; HOMA-IR: Homeostatic Model Assessment for Insulin Resistance.

**Figure 2:**
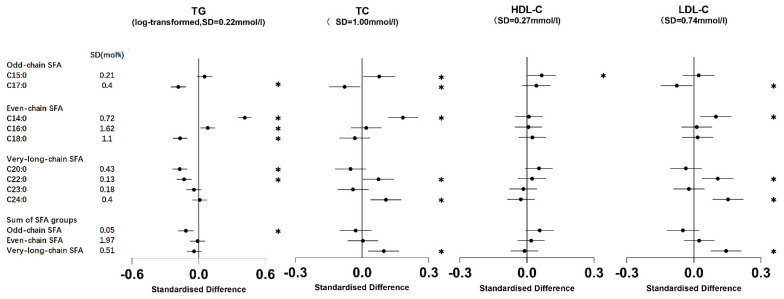
Associations of erythrocyte membrane saturated fatty acids (SFAs) with triglycerides, total cholesterol, and types of cholesterol. The standardized difference represents the change (in standard deviation units of the metabolic marker) per one standard deviation change in each SFA. The error bars depict 95% confidence intervals (CI). * signifies that the association had a *p*-value of <0.05. Models were adjusted for age, sex, BMI, smoking status, alcohol drinking, physical activity, family history of diseases, and total energy intake; TG was log-transformed. TC: total cholesterol; HDL-C: high-density lipoprotein cholesterol; LDL-C: low-density lipoprotein cholesterol.

**Figure 3:**
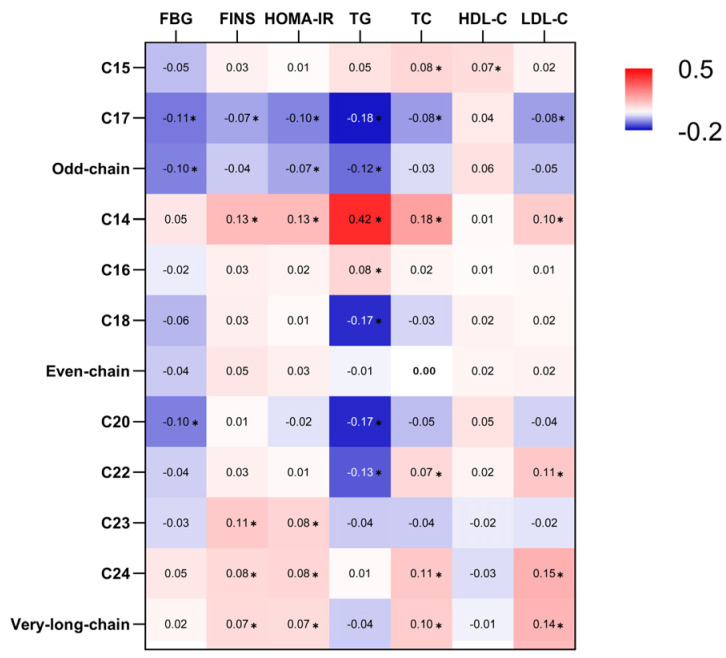
Summary of associations between erythrocyte membrane saturated fatty acids (SFAs) and metabolic markers. Each cell represents the standardized difference (in SD units) of metabolic markers per one SD difference in erythrocyte SFAs. Values with * indicate that the *p* value for the association was <0.05. All values are depicted on a color scale, with red indicating varying degrees of positive associations and blue indicating varying degrees of negative associations.

C15:0 exhibited a positive association between TC and HDL-C. C17:0 showed a negative association with most metabolic markers, with no evidence of an association with HDL-C. The odd-chain SFA group (sum of C15:0 and C17:0) displayed negative associations with FBG, HOMA-IR, and TG (Figure 1, Figure 2 and Figure 3, Table S2).

#### 3.2.2. Even-Chain SFAs

Both C14:0 and C16:0 exhibited positive associations with TG. In addition, C14:0 was positively associated with FINS, HOMA-IR, TC, and LDL-C. Conversely, C18:0 demonstrated an inverse association with TG (Figure 1, Figure 2 and Figure 3, Table S3). There was no evidence to suggest an association between the even-chain group and metabolic markers (Figure 3).

#### 3.2.3. Very-Long-Chain SFAs

C20:0 exhibited an inverse association with FBG and TG. C22:0 displayed an inverse association with TG. Both C22:0 and C24:0 exhibited positive associations with TC and LDL-C. In addition, C23:0 and C24:0 exhibited positive associations with FINS and HOMA-IR (Figure 1, Figure 2 and Figure 3, Table S4). The very-long-chain SFA group was associated with metabolic markers similar to those observed for C24:0 (Figure 3).

### 3.3. Sensitivity and Interaction Analyses

The majority of the sensitivity analyses had no substantial effect on the results (Table S5). Results from interaction analyses revealed that odd-chain SFAs and very-long-chain SFAs significantly interacted with alcohol drinking, while odd-chain SFAs interacted with sex (Figure S1). Specifically, the association of the odd-chain SFA group with TG was stronger among alcohol drinkers compared to non-alcohol drinkers. No association was observed between the odd-chain SFA group and HDL-C in alcohol drinkers, while a positive association was found in non-alcohol drinkers. Conversely, the very-long-chain SFA group demonstrated a significant inverse association with HDL-C among alcohol drinkers. In addition, there was an inverse association observed between the odd-chain SFA group and TC among men but not women.

## 4. Discussion

In this study, we investigated the associations between nine individual erythrocyte membrane SFAs and several groups of SFAs with seven metabolic markers. We found different associations between SFAs and metabolic markers. Our results indicated favorable associations between the odd-chain SFA group and glycemic and lipid metabolic markers. Even-chain SFA groups had no associations with metabolic markers. Additionally, positive associations were observed between the very-long-chain SFA group and glycemic and lipid metabolic markers.

Fatty acids play a crucial role in maintaining the fluidity and functionality of cell membranes and are intricately associated with glycolipid metabolism [15,16,17]. The glycemic markers (FBG, FINS, and HOMA-IR) are positively associated with insulin resistance and represent significant risk factors for CVD [18]. The abnormal levels of lipid markers, including elevated levels of LDL-C, TC, and TG, along with decreased HDL-C, are well-established indicators of lipid metabolism and significant risk factors for CVD [19,20].

Odd-chain SFAs demonstrated favorable associations with glycemic and lipid profiles, suggesting potential benefits for reducing cardiovascular risks. C15:0 displayed favorable associations with HDL-C levels, while C17:0 exhibited inverse associations with various metabolic markers (FBG, FINS, HOMA-IR, TG, TC, and LDL-C). These findings support existing evidence linking odd-chain SFAs to improved metabolic and cardiovascular health risks [8,21]. When considering the combined effects of the odd-chain SFA group (sum of C15:0 and C17:0), we observed negative associations with FBG, HOMA-IR, and TC levels, suggesting potential benefits in reducing cardiovascular risks. These results align with previous studies that have linked odd-chain SFAs to a reduction in metabolic and CVD risks [7,22,23,24]. However, in contrast to the plasma phospholipid odd-chain SFA group in the EPIC study, we found no association between the erythrocyte membrane odd-chain SFA group and lipid markers such as TC and HDL-C [7]. One possible explanation for this discrepancy is the use of different lipid fractions for analysis. Additionally, the proportion of odd-chain SFAs in total SFAs of the erythrocyte membrane in our study is lower than in Western countries [25], potentially making it difficult to observe correlations with lipid markers.

In our study, we observed a positive association between C14:0 and C16:0 and the metabolic markers of CVD, aligning with previous research linking even-chain SFAs to adverse metabolic risks [4,7,14]. However, we observed an unexpected inverse association between C18:0 and TG, contradicting some previous findings [7,14,26]. However, a recent meta-analysis found no association between C18:0 and CVD or CHD [8], and another investigation suggested that C18:0 has no significant impact on lipid metabolism [27]. The conflicting evidence on C18:0 and its association with metabolic health necessitates further investigation. Additionally, we found no association between the even-chain SFA group and metabolic markers, which contradicted prior western evidence in plasma phospholipid SFAs [7]. However, an independent prospective cohort study conducted in China reported no association between the even-chain SFA group and T2D but found positive associations with individual SFAs [26]. The differences between Western and Asian populations in dietary patterns and nutritional compositions may explain these inconsistencies [28]. Previous studies mainly involved Western populations with higher consumption of high-fat products rich in SFAs, particularly even-chain SFAs, compared to Asian populations [29,30,31]. Furthermore, differences in fatty acid measurement across various blood components may also contribute to inconsistent findings. Research indicates a stronger association for fatty acids measured in plasma phospholipids compared to erythrocytes, potentially explaining our overall lack of significant results regarding the even-chain SFA group [26,32].

Our study found positive associations between the very-long-chain SFAs and atherogenic lipid markers, such as TC and LDL-C, consistent with prior studies on lipid marker levels [7,33]. C20:0 and C22:0 exhibited an inverse association with TG, consistent with the observed direction in CVD and T2D risks [34,35,36,37]. Moreover, elevated levels of lipid markers (TC and LDL-C) were linked with high levels of C22:0 and C24:0, in alignment with the findings of the EPIC cross-sectional study [7], and C23:0 and C24:0 exhibited positive associations with insulin resistance markers (FINS and HOMA-IR). These findings on metabolic markers were in contrast with studies suggesting that higher levels of very-long-chain SFAs are associated with a lower incidence of T2D and CVD [34,37]. Given these contradictory mechanistic results, further investigations are necessary to explore the reasons for the inconsistency and to elucidate the underlying mechanisms.

In our study, we identified interactions among alcohol consumption, sex, and SFAs exerting influence on metabolic markers. Specifically, we noted consistent interaction patterns in the stratified analysis for alcohol between the odd-chain SFA group and TG, particularly among individuals who reported alcohol consumption. Non-alcohol drinkers showed a positive association between the very-long-chain SFA group and HDL-C levels. These findings suggest the need for further research to explore the impact of alcohol consumption quantity on SFAs and metabolic markers. Moreover, we observed an inverse association between the odd-chain SFA group and TC levels in males, whereas this association was absent in females. These findings are in line with results from the EPIC study [7]. These interactions suggested that alcohol consumption and sex should be taken into account in evaluating the relationship between individual SFAs and metabolic risk factors.

The results of our study established a potential link between SFAs and the risk of CVD through markers of glycemic and lipid metabolism. Initially, our investigation revealed a favorable association between odd-chain SFAs and markers related to glycemic and lipid profiles. We hypothesized that this association might contribute to the inverse relationship between odd-chain SFAs and CVD risks. Secondly, the even-chain SFA group demonstrated no association with glycemic and lipid markers in our study, but C14:0 particularly showed a notable positive correlation with several key markers. A comprehensive systematic meta-analysis [38] incorporating 51 studies with 1526 participants revealed that C14:0 had a more significant impact on cholesterol ratios compared to C16:0 and C18:0. In addition, clinical evidence has suggested its potent cholesterol-upregulating action [39]. We speculate that C14:0 may be related to lipid metabolism pathways, and its potential biological mechanisms are worthy of further investigation. Thirdly, when discussing the connections between phospholipid very-long-chain SFAs and physiologic risks, it is crucial to acknowledge their highly specialized function [40]. Very-long-chain SFAs are almost exclusively found in sphingolipids and ceramides. Ceramides have been considered important contributors to insulin resistance [41]. Ceramide inhibits insulin signaling and mediates the extrinsic apoptotic pathway, ultimately leading to reduced insulin synthesis [42,43]. Although our findings indicated a positive association between the very-long-chain SFA group and markers of glycolipid metabolic markers, many studies support a negative association with CVD and T2D risks [34,37,44]. The very-long-chain SFAs, specifically C20:0 and C22:0, exhibit favorable associations with TG, which may contribute to the mechanisms underlying the inverse relationship with CVD [45]. Additionally, we observed a cross-sectional association between very-long-chain SFAs and elevated levels of LDL-C, in alignment with previous research [7,33]. Sphingomyelins in cell membranes tend to cluster in specialized lipid rafts rich in cholesterol [46]. Therefore, the observed connection between very-long-chain SFAs and LDL-C levels may signify the inherent association between sphingomyelins and cholesterol within membranes rather than indicating a pathological mechanism.

Our study exhibits several strengths. Firstly, we employed an objective measurement of erythrocyte membrane SFAs. Secondly, we performed multiple stratified analyses and sensitivity analyses to strengthen the robustness of our findings. These analyses allow us to assess the consistency and stability of our results under different methodological assumptions and statistical models, thereby increasing our confidence in the reliability of our results.

Several limitations should be considered. Firstly, our study adopts a cross-sectional design, thereby constraining our capacity to establish causal relationships between erythrocyte membrane SFAs and metabolic markers. Secondly, the small sample size of our study population may limit the generalizability of our findings to broader populations. Thirdly, it is important to acknowledge that our study involved multiple analyses, which may increase the likelihood of false-positive results. However, given the exploratory nature of our investigation with a limited sample size, our focus was on maintaining a degree of openness and adaptability. We opted against correcting for multiple testing to prevent excessive penalization of potential discoveries, thereby preserving opportunities for identifying promising associations. Nonetheless, it is plausible that some of our observed findings may stem from chance rather than genuine correlations, necessitating cautious interpretation. Further research endeavors should leverage larger sample sizes, employ more stringent methodologies to authenticate our findings, and delve deeper into potential mechanisms. Furthermore, our analysis may be limited by the restricted number of parameters that could influence the results, including the absence of adjustments to renal function, serum uric acid levels, and drug use. Additionally, the FFQ questionnaire’s limitations may introduce inherent biases in dietary data, stemming from participant recall and inaccuracies in quantifying food intake. These confounding factors have the potential to impact the outcomes of our study.

## 5. Conclusions

Our analysis indicates that odd-chain SFAs exhibit an inverse correlation with various metabolic markers involved in glycemic and lipid pathways, potentially contributing to their inverse associations with CVD. However, the relationship between even-chain SFAs and markers of glycemic and lipid metabolism requires further investigation. Similarly, the positive associations of very-long-chain SFAs with glycemic and lipid markers warrant additional study. Furthermore, our investigation reveals interactions among alcohol, sex, and SFAs on metabolic risks, which introduce complexity to the relationship between SFAs and cardiovascular risks. Overall, these findings contribute to a deeper understanding of the association between SFAs and cardiovascular risks, offering valuable insights for researchers and health professionals. In summary, these findings enhance our comprehension of the role played by SFAs in metabolic pathways and the genesis of CVD. Nonetheless, additional investigations are needed to validate our findings and elucidate the mechanisms underlying these associations. By delving deeper into the role of SFAs in metabolic processes and the origins of CVD, we can more effectively guide preventive strategies and interventions aimed at diminishing cardiovascular risk and improving overall public health.

## Figures and Tables

**Table 1:** Baseline characteristics of the study population (*n* = 798).

Baseline Characteristics	Mean±SD/*n* (%)
Age (year)	55.12 ± 7.75
Sex	
Women	547 (68.5)
Men	251 (31.5)
Weight (kg)	62.75 ± 10.43
Height (cm)	159.76 ± 8.12
Body mass index, BMI (kg/m^2^)	
<24	356 (44.6)
24–28	336 (42.1)
>28	106 (13.3)
Waist circumference (cm)	85.57 ± 8.91
Smoker	
No	700 (87.7)
Yes	98 (12.3)
Alcohol drinker	
No	669 (83.8)
Yes	129 (16.2)
Physical activity	
Light	282 (35.3)
Moderate	379 (47.5)
Vigorous	137 (17.2)
Family history of diseases	
No	652 (81.7)
Yes	146 (18.3)
Total energy intake (kcal/day)	1648 ± 468
Dairy intake (g/day)	90.18 ± 99.82
Red and processed meat intake (g/day)	78.90 ± 55.65
Fruits and vegetables intake (g/day)	412.09 ± 212.76
Vegetable oil intake (g/day)	32.60 ± 21.37

**Table 2:** Distribution of serum metabolic biomarkers and erythrocyte membrane saturated fatty acid groups by sex, age, and BMI in the research.

	Overall	Sex			Age (Years)			BMI (kg/m^2^)			
	*n* = 798	Men (*n* = 251)	Women (*n* = 547)	*p*	≤65 (*n* = 716)	>65 (*n* = 82)	*p*	<24 (*n* = 355)	24–28 (*n* = 340)	>28 (*n* = 103)	*p*
FBG (mmol/L) ^a^	5.50 (5.25, 5.89)	5.48 (5.19, 5.86)	5.47 (5.18, 5.83)	0.337	5.46 (5.18, 5.84)	5.58 (5.20, 5.85)	0.353	5.36 (5.11, 5.67)	5.57 (5.22, 5.98)	5.62 (5.27, 6.03)	<0.001
FINS (μU/mL) ^a^	6.92 (6.03, 8.32)	6.91 (5.89, 8.20)	6.85 (5.99, 8.41)	0.793	6.85 (5.96, 8.31)	6.86 (5.99, 8.49)	0.815	6.48 (5.78, 7.67)	7.31 (6.16, 8.70)	7.39 (6.56, 9.91)	<0.001
HOMA-IR ^a^	1.74 (1.41, 2.14)	1.76 (1.42, 2.11)	1.71 (1.41, 2.14)	0.700	1.72 (1.41, 2.12)	1.77 (1.44, 2.14)	0.502	1.54 (1.35, 1.86)	1.81 (1.48, 2.27)	1.92 (1.63, 2.71)	<0.001
TG (mmol/L) ^a^	1.26 (0.91, 1.78)	1.48 (1.03, 2.11)	1.17 (0.88, 1.67)	<0.001	1.25 (0.91, 1.77)	1.35 (0.94, 1.72)	0.757	1.13 (0.83, 1.52)	1.36 (0.96, 2.03)	1.48 (1.08, 1.94)	<0.001
TC (mmol/L) ^b^	5.48 (1.00)	5.36 (0.88)	5.54 (1.04)	0.047	5.49 (1.01)	5.38 (0.93)	0.403	5.55 (1.00)	5.45 (0.98)	5.35 (1.03)	0.087
HDL-C (mmol/L) ^b^	1.29 (0.27)	1.15 (0.23)	1.36 (0.27)	<0.001	1.29 (0.27)	1.28 (0.28)	0.812	1.39 (0.27)	1.23 (0.24)	1.16 (0.22)	<0.001
LDL-C (mmol/L) ^b^	3.45 (0.74)	3.45 (0.66)	3.46 (0.78)	0.934	3.46 (0.75)	3.42 (0.72)	0.675	3.45 (0.75)	3.46 (0.74)	3.44 (0.74)	0.824
Odd-chain SFAs (mol%) ^b^	0.36 (0.05)	0.34 (0.05)	0.36 (0.05)	<0.001	0.36 (0.06)	0.36 (0.05)	0.861	0.37 (0.05)	0.35 (0.05)	0.34 (0.05)	<0.001
C15:0 (mol%) ^b^	0.08 (0.02)	0.08 (0.02)	0.09 (0.02)	<0.001	0.08 (0.02)	0.08 (0.02)	0.302	0.09 (0.02)	0.08 (0.02)	0.08 (0.02)	<0.001
C17:0 (mol%) ^b^	0.27 (0.04)	0.27 (0.04)	0.28 (0.04)	<0.001	0.27 (0.04)	0.27 (0.04)	0.969	0.28 (0.04)	0.27 (0.04)	0.26 (0.04)	<0.001
Even-chain SFAs (mol%) ^b^	44.34 (1.97)	44.27 (2.04)	44.37 (1.95)	0.446	44.37 (1.96)	44.04 (2.06)	0.038	44.30 (1.99)	44.37 (1.96)	44.35 (1.96)	0.993
C14:0 (mol%) ^b^	0.25 (0.07)	0.24 (0.07)	0.26 (0.07)	0.014	0.25 (0.07)	0.26 (0.07)	0.624	0.25 (0.07)	0.25 (0.07)	0.26 (0.07)	0.086
C16:0 (mol%) ^b^	31.40 (1.62)	31.44 (1.70)	31.40 (1.58)	0.934	31.43 (1.62)	31.26 (1.60)	0.205	31.42 (1.67)	31.40 (1.60)	31.42 (1.46)	0.973
C18:0 (mol%) ^b^	12.67 (1.10)	12.59 (1.03)	12.71 (1.13)	0.215	12.69 (1.11)	12.52 (1.00)	0.149	12.63 (1.12)	12.72 (1.10)	12.66 (1.07)	0.674
Very-long-chain SFAs (mol%) ^b^	3.85 (0.51)	3.80 (0.50)	3.88 (0.51)	0.106	3.85 (0.51)	3.88 (0.46)	0.456	3.83 (0.51)	3.87 (0.52)	3.91 (0.44)	0.205
C20:0 (mol%) ^b^	0.24 (0.04)	0.23 (0.03)	0.25 (0.05)	<0.001	0.24 (0.04)	0.24 (0.04)	0.735	0.24 (0.04)	0.24 (0.04)	0.24 (0.04)	0.574
C22:0 (mol%) ^b^	0.85 (0.13)	0.81 (0.12)	0.86 (0.13)	<0.001	0.85 (0.14)	0.85 (0.11)	0.740	0.85 (0.13)	0.84 (0.14)	0.84 (0.11)	0.571
C23:0 (mol%) ^b^	0.09 (0.02)	0.08 (0.02)	0.09 (0.02)	<0.001	0.09 (0.02)	0.08 (0.02)	0.015	0.09 (0.02)	0.08 (0.02)	0.09 (0.02)	0.298
C24:0 (mol%) ^b^	2.68 (0.40)	2.68 (0.40)	2.68 (0.39)	0.624	2.68 (0.40)	2.71 (0.36)	0.346	2.65 (0.40)	2.70 (0.40)	2.74 (0.36)	0.044

^a^ metabolic markers were presented as median (interquartile range); ^b^ metabolic markers or SFA groups were presented as mean (SD). FBG: fasting blood glucose; FINS: fasting insulins; HOMA-IR: Homeostatic Model Assessment for Insulin Resistance; TG: triglycerides; TC: total cholesterol; HDL-C: high-density lipoprotein cholesterol; LDL-C: low-density lipoprotein cholesterol; SFA: saturated fatty acid.

## Data Availability

The original contributions presented in the study are included in the article/Supplementary Material, further inquiries can be directed to the corresponding authors.

## Supplementary Materials

The following supporting information can be downloaded at: https://www.mdpi.com/article/10.3390/nu16101507/s1. Table S1. Spearman’s rank correlation of erythrocyte membrane saturated fatty acids (*n* = 798); Table S2. Association of erythrocyte membrane odd-chain saturated fatty acids (per one SD difference) with metabolic markers; Table S3. Association of erythrocyte membrane even-chain saturated fatty acids (per one SD difference) with metabolic markers; Table S4. Association of erythrocyte membrane very-long-chain saturated fatty acids with (per one SD difference) with metabolic markers; Table S5. Sensitivity analysis for the association of erythrocyte membrane saturated fatty acid groups and metabolic markers; Figure S1. Association of erythrocyte membrane saturated fatty acid groups with metabolic markers by subgroups of alcohol intake and sex.

## References

[1] Younus A., Aneni E.C., Spatz E.S., Osondu C.U., Roberson L., Ogunmoroti O., Malik R., Ali S.S., Aziz M., Feldman T. (2016). A Systematic Review of the Prevalence and Outcomes of Ideal Cardiovascular Health in US and Non-US Populations. Mayo Clin. Proc..

[2] (2022). Report on Cardiovascular Health and Diseases in China 2021: An Updated Summary. Biomed. Environ. Sci. BES.

[3] Arnett D.K., Blumenthal R.S., Albert M.A., Buroker A.B., Goldberger Z.D., Hahn E.J., Himmelfarb C.D., Khera A., Lloyd-Jones D., McEvoy J.W. (2019). 2019 ACC/AHA Guideline on the Primary Prevention of Cardiovascular Disease: Executive Summary: A Report of the American College of Cardiology/American Heart Association Task Force on Clinical Practice Guidelines. Circulation.

[4] Khaw K.T., Friesen M.D., Riboli E., Luben R., Wareham N. (2012). Plasma phospholipid fatty acid concentration and incident coronary heart disease in men and women: The EPIC-Norfolk prospective study. PLoS Med..

[5] Lemaitre R.N., King I.B., Rice K., McKnight B., Sotoodehnia N., Rea T.D., Johnson C.O., Raghunathan T.E., Cobb L.A., Mozaffarian D. (2014). Erythrocyte very long-chain saturated fatty acids associated with lower risk of incident sudden cardiac arrest. Prostaglandins Leukot. Essent. Fat. Acids.

[6] Lemaitre R.N., McKnight B., Sotoodehnia N., Fretts A.M., Qureshi W.T., Song X., King I.B., Sitlani C.M., Siscovick D.S., Psaty B.M. (2018). Circulating Very Long-Chain Saturated Fatty Acids and Heart Failure: The Cardiovascular Health Study. J. Am. Heart Assoc..

[7] Zheng J.S., Sharp S.J., Imamura F., Koulman A., Schulze M.B., Ye Z., Griffin J., Guevara M., Huerta J.M., Kröger J. (2017). Association between plasma phospholipid saturated fatty acids and metabolic markers of lipid, hepatic, inflammation and glycaemic pathways in eight European countries: A cross-sectional analysis in the EPIC-InterAct study. BMC Med..

[8] Li Z., Lei H., Jiang H., Fan Y., Shi J., Li C., Chen F., Mi B., Ma M., Lin J. (2022). Saturated fatty acid biomarkers and risk of cardiometabolic diseases: A meta-analysis of prospective studies. Front. Nutr..

[9] Kirk E.P., Klein S. (2009). Pathogenesis and pathophysiology of the cardiometabolic syndrome. J. Clin. Hypertens..

[10] Ainsworth B.E., Haskell W.L., Whitt M.C., Irwin M.L., Swartz A.M., Strath S.J., O’Brien W.L., Bassett D.R., Schmitz K.H., Emplaincourt P.O. (2000). Compendium of physical activities: An update of activity codes and MET intensities. Med. Sci. Sports Exerc..

[11] Zhang B., Wang P., Chen C.G., He Q.Q., Zhuo S.Y., Chen Y.M., Su Y.X. (2010). Validation of an FFQ to estimate the intake of fatty acids using erythrocyte membrane fatty acids and multiple 3d dietary records. Public Health Nutr..

[12] Yang Y., Wang G., Pan X. (2009). China Food Composition.

[13] Su M., Zhang X., Hu W., Yang Z., Chen D., Yang Y., Xie K., Chen Y., Zhang Z. (2023). The associations of erythrocyte membrane polyunsaturated fatty acids with skeletal muscle loss: A prospective cohort study. Clin. Nutr..

[14] Forouhi N.G., Koulman A., Sharp S.J., Imamura F., Kröger J., Schulze M.B., Crowe F.L., Huerta J.M., Guevara M., Beulens J.W. (2014). Differences in the prospective association between individual plasma phospholipid saturated fatty acids and incident type 2 diabetes: The EPIC-InterAct case-cohort study. Lancet Diabetes Endocrinol..

[15] Zheng J.S., Lin J.S., Dong H.L., Zeng F.F., Li D., Song Y., Chen Y.M. (2019). Association of erythrocyte n-3 polyunsaturated fatty acids with incident type 2 diabetes in a Chinese population. Clin. Nutr..

[16] Pikó P., Pál L., Szűcs S., Kósa Z., Sándor J., Ádány R. (2021). Obesity-Related Changes in Human Plasma Lipidome Determined by the Lipidyzer Platform. Biomolecules.

[17] Riccardi G., Giacco R., Rivellese A.A. (2004). Dietary fat, insulin sensitivity and the metabolic syndrome. Clin. Nutr..

[18] González-González J.G., Violante-Cumpa J.R., Zambrano-Lucio M., Burciaga-Jimenez E., Castillo-Morales P.L., Garcia-Campa M., Solis R.C., González-Colmenero A.D., Rodríguez-Gutiérrez R. (2022). HOMA-IR as a predictor of Health Outcomes in Patients with Metabolic Risk Factors: A Systematic Review and Meta-analysis. High Blood Press. Cardiovasc. Prev. Off. J. Ital. Soc. Hypertens..

[19] Miller M., Stone N.J., Ballantyne C., Bittner V., Criqui M.H., Ginsberg H.N., Goldberg A.C., Howard W.J., Jacobson M.S., Kris-Etherton P.M. (2011). Triglycerides and cardiovascular disease: A scientific statement from the American Heart Association. Circulation.

[20] Mach F., Baigent C., Catapano A.L., Koskinas K.C., Casula M., Badimon L., Chapman M.J., De Backer G.G., Delgado V., Ference B.A. (2020). 2019 ESC/EAS Guidelines for the management of dyslipidaemias: Lipid modification to reduce cardiovascular risk. Eur. Heart J..

[21] Huang L., Lin J.S., Aris I.M., Yang G., Chen W.Q., Li L.J. (2019). Circulating Saturated Fatty Acids and Incident Type 2 Diabetes: A Systematic Review and Meta-Analysis. Nutrients.

[22] de Oliveira Otto M.C., Nettleton J.A., Lemaitre R.N., Steffen L.M., Kromhout D., Rich S.S., Tsai M.Y., Jacobs D.R., Mozaffarian D. (2013). Biomarkers of dairy fatty acids and risk of cardiovascular disease in the Multi-ethnic Study of Atherosclerosis. J. Am. Heart Assoc..

[23] Imamura F., Fretts A., Marklund M., Ardisson Korat A.V., Yang W.S., Lankinen M., Qureshi W., Helmer C., Chen T.A., Wong K. (2018). Fatty acid biomarkers of dairy fat consumption and incidence of type 2 diabetes: A pooled analysis of prospective cohort studies. PLoS Med..

[24] Chowdhury R., Warnakula S., Kunutsor S., Crowe F., Ward H.A., Johnson L., Franco O.H., Butterworth A.S., Forouhi N.G., Thompson S.G. (2014). Association of dietary, circulating, and supplement fatty acids with coronary risk: A systematic review and meta-analysis. Ann. Intern. Med..

[25] Jacobs S., Schiller K., Jansen E., Fritsche A., Weikert C., di Giuseppe R., Boeing H., Schulze M.B., Kröger J. (2015). Association between erythrocyte membrane fatty acids and biomarkers of dyslipidemia in the EPIC-Potsdam study. Eur. J. Clin. Nutr..

[26] Lin J.S., Dong H.L., Chen G.D., Chen Z.Y., Dong X.W., Zheng J.S., Chen Y.M. (2018). Erythrocyte Saturated Fatty Acids and Incident Type 2 Diabetes in Chinese Men and Women: A Prospective Cohort Study. Nutrients.

[27] Flock M.R., Kris-Etherton P.M. (2013). Diverse physiological effects of long-chain saturated fatty acids: Implications for cardiovascular disease. Curr. Opin. Clin. Nutr. Metab. Care.

[28] Sun L., Zong G., Li H., Lin X. (2021). Fatty acids and cardiometabolic health: A review of studies in Chinese populations. Eur. J. Clin. Nutr..

[29] Sebastian R.S., Enns C.W., Martin C.L., Goldman J.D., Moshfegh A.J. (2010). Sweet Foods Consumption by Adults in the U.S. What We Eat in America, NHANES 2015–2018. FSRG Dietary Data Briefs.

[30] Leme A.C., Baranowski T., Thompson D., Philippi S., O’Neil C., Fulgoni V., Nicklas T. (2019). Top food sources of percentage of energy, nutrients to limit and total gram amount consumed among US adolescents: National Health and Nutrition Examination Survey 2011–2014. Public Health Nutr..

[31] Zhao R., Zhao L., Gao X., Yang F., Yang Y., Fang H., Ju L., Xu X., Guo Q., Li S. (2022). Geographic Variations in Dietary Patterns and Their Associations with Overweight/Obesity and Hypertension in China: Findings from China Nutrition and Health Surveillance (2015–2017). Nutrients.

[32] Patel P.S., Sharp S.J., Jansen E., Luben R.N., Khaw K.T., Wareham N.J., Forouhi N.G. (2010). Fatty acids measured in plasma and erythrocyte-membrane phospholipids and derived by food-frequency questionnaire and the risk of new-onset type 2 diabetes: A pilot study in the European Prospective Investigation into Cancer and Nutrition (EPIC)-Norfolk cohort. Am. J. Clin. Nutr..

[33] Lemaitre R.N., Fretts A.M., Sitlani C.M., Biggs M.L., Mukamal K., King I.B., Song X., Djoussé L., Siscovick D.S., McKnight B. (2015). Plasma phospholipid very-long-chain saturated fatty acids and incident diabetes in older adults: The Cardiovascular Health Study. Am. J. Clin. Nutr..

[34] Fretts A.M., Imamura F., Marklund M., Micha R., Wu J.H.Y., Murphy R.A., Chien K.L., McKnight B., Tintle N., Forouhi N.G. (2019). Associations of circulating very-long-chain saturated fatty acids and incident type 2 diabetes: A pooled analysis of prospective cohort studies. Am. J. Clin. Nutr..

[35] Tao X., Liu L., Ma P., Hu J., Ming Z., Dang K., Zhang Y., Li Y. (2023). Association of Circulating Very Long Chain Saturated Fatty Acids with Cardiovascular Mortality in NHANES 2015–2016. J. Clin. Endocrinol. Metab..

[36] Matsumori R., Miyazaki T., Shimada K., Kume A., Kitamura Y., Oshida K., Yanagisawa N., Kiyanagi T., Hiki M., Fukao K. (2013). High levels of very long-chain saturated fatty acid in erythrocytes correlates with atherogenic lipoprotein profiles in subjects with metabolic syndrome. Diabetes Res. Clin. Pract..

[37] Liu M., Zuo L.S., Sun T.Y., Wu Y.Y., Liu Y.P., Zeng F.F., Chen Y.M. (2020). Circulating Very-Long-Chain Saturated Fatty Acids Were Inversely Associated with Cardiovascular Health: A Prospective Cohort Study and Meta-Analysis. Nutrients.

[38] Fattore E., Bosetti C., Brighenti F., Agostoni C., Fattore G. (2014). Palm oil and blood lipid-related markers of cardiovascular disease: A systematic review and meta-analysis of dietary intervention trials. Am. J. Clin. Nutr..

[39] Bradbury K.E., Skeaff C.M., Green T.J., Gray A.R., Crowe F.L. (2010). The serum fatty acids myristic acid and linoleic acid are better predictors of serum cholesterol concentrations when measured as molecular percentages rather than as absolute concentrations. Am. J. Clin. Nutr..

[40] Lauritzen L., Hellgren L.I. (2015). Plasma phospholipid very-long-chain saturated fatty acids: A sensitive marker of metabolic dysfunction or an indicator of specific healthy dietary components?. Am. J. Clin. Nutr..

[41] Chavez J.A., Summers S.A. (2012). A ceramide-centric view of insulin resistance. Cell Metab..

[42] Chandra J., Zhivotovsky B., Zaitsev S., Juntti-Berggren L., Berggren P.O., Orrenius S. (2001). Role of apoptosis in pancreatic beta-cell death in diabetes. Diabetes.

[43] Véret J., Coant N., Berdyshev E.V., Skobeleva A., Therville N., Bailbé D., Gorshkova I., Natarajan V., Portha B., Le Stunff H. (2011). Ceramide synthase 4 and de novo production of ceramides with specific N-acyl chain lengths are involved in glucolipotoxicity-induced apoptosis of INS-1 β-cells. Biochem. J..

[44] Ardisson Korat A.V., Malik V.S., Furtado J.D., Sacks F., Rosner B., Rexrode K.M., Willett W.C., Mozaffarian D., Hu F.B., Sun Q. (2020). Circulating Very-Long-Chain SFA Concentrations Are Inversely Associated with Incident Type 2 Diabetes in US Men and Women. J. Nutr..

[45] Lemaitre R.N., King I.B. (2022). Very long-chain saturated fatty acids and diabetes and cardiovascular disease. Curr. Opin. Lipidol..

[46] Hanzal-Bayer M.F., Hancock J.F. (2007). Lipid rafts and membrane traffic. FEBS Lett..

