# Supplementary material for: Differential Associations of Erythrocyte Membrane Saturated Fatty Acids with Glycemic and Lipid Metabolic Markers in a Chinese Population: A Cross-Sectional Study
Source: Nutrients. 2024 May 16;16(10):1507. doi: 10.3390/nu16101507 (PMC11123842; doi:10.3390/nu16101507)
Supplement: Supplementary file 1 [file nutrients-16-01507-s001.zip › nutrients-2969841-supplementary.pdf]

Table S1 Spearman's rank correlation of erythrocyte membrane saturated fatty acids (n = 798) [a]

	C15:0	C17:0	Odd-chain SFA	C14:0	C16:0	C18:0	Even-chain SFA	C20:0	C22:0	C23:0	C24:0	Very-long-chain SFA
C15:0	1.000											
C17:0	0.537**	1.000										
Odd-chain SFA	0.785**	0.937**	1.000									
C14:0	0.527**	-0.018	0.192**	1.000								
C16:0	0.155**	-0.030	0.033	0.355**	1.000							
C18:0	-0.199**	0.031	-0.062	-0.242**	0.051	1.000						
Even-chain SFA	0.080*	0.018	0.037	0.237**	0.851**	0.511**	1.000					
C20:0	0.078*	0.210**	0.179**	-0.033	0.082*	0.323**	0.245**	1.000				
C22:0	0.145**	0.125**	0.145**	0.032	-0.151**	-0.015	-0.105**	0.421**	1.000			
C23:0	0.268**	0.300**	0.323**	-0.003	0.067	0.194**	0.162**	0.185**	0.242**	1.000		
C24:0	0.052	-0.006	0.017	0.074*	-0.163**	-0.154**	-0.184**	0.155**	0.611**	0.110**	1.000	
Very-long-chain SFA	0.093**	0.055	0.077*	0.064	-0.162**	-0.096**	-0.152**	0.315**	0.792**	0.207**	0.955**	1.000

[a] Spearman's rank correlation coefficients were calculated between individual erythrocyte saturated fatty acids; * $P < 0.05$, ** $P < 0.01$,*** $P < 0.001$.

Table S2 Association of erythrocyte membrane odd-chain saturated fatty acids (per 1 SD difference) with metabolic markers

		C15:0	C17:0	Odd-chain SFA
	Model	Standardised Difference (CI) [b]	Standardised Difference (CI) [b]	Standardised Difference (CI) [b]
FBG (mmol/L)[a]	Model 1	-0.049(-0.118,0.020)	-0.149(-0.217,-0.080)	-0.128(-0.197,-0.059)
	Model 2	-0.034(-0.104,0.036)	-0.112(-0.181,-0.043)	-0.097(-0.167,-0.027)
	Model 3	-0.052(-0.122,0.017)	-0.106(-0.175,-0.038)**	-0.099(-0.169,-0.030)**
FINS (μU/ml)[a]	Model 1	-0.004(-0.073,0.066)	-0.134(-0.203,-0.065)	-0.099(-0.169,-0.030)
	Model 2	0.033(-0.036,0.103)	-0.079(-0.148,-0.010)	-0.046(-0.116,0.024)
	Model 3	0.027(-0.043,0.097)	-0.071(-0.140,-0.002)*	-0.042(-0.112,0.027)
HOMA-IR[a]	Model 1	-0.019(-0.089,0.050)	-0.166(-0.235,-0.098)	-0.129(-0.198,-0.060)
	Model 2	0.018(-0.050,0.087)	-0.106(-0.174,-0.038)	-0.072(-0.141,-0.003)
	Model 3	0.007(-0.062,0.076)	-0.097(-0.165,-0.030)**	-0.07(-0.138,-0.001)*
TG (mmol/L)[a]	Model 1	-0.004(-0.073,0.066)	-0.246(-0.314,-0.179)	-0.181(-0.250,-0.113)
	Model 2	0.056(-0.014,0.126)	-0.195(-0.263,-0.127)	-0.123(-0.193,-0.054)
	Model 3	0.054(-0.016,0.123)	-0.183(-0.250,-0.115)***	-0.115(-0.184,-0.046)**
TC (mmol/L)	Model 1	0.111(0.041,0.180)	-0.053(-0.122,0.017)	0.005(-0.065,0.074)
	Model 2	0.075(0.004,0.147)	-0.084(-0.155,-0.014)	-0.033(-0.105,0.038)
	Model 3	0.077(0.005,0.149)*	-0.079(-0.150,-0.008)*	-0.029(-0.101,0.043)
HDL-C (mmol/L)	Model 1	0.182(0.114,0.251)	0.170(0.101,0.239)	0.195(0.127,0.263)
	Model 2	0.062(-0.002,0.125)	0.051(-0.012,0.114)	0.062(-0.002,0.126)
	Model 3	0.066(0.002,0.130)*	0.042(-0.021,0.106)	0.057(-0.007,0.121)
LDL-C (mmol/L)	Model 1	0.036(-0.034,0.105)	-0.077(-0.146,-0.007)	-0.042(-0.112,0.027)
	Model 2	0.021(-0.050,0.093)	-0.083(-0.154,-0.012)	-0.053(-0.125,0.018)
	Model 3	0.022(-0.051,0.094)	-0.076(-0.148,-0.005)*	-0.049(-0.121,0.024)

[a] FBG,FINS,HOMA-IR,TG were log-transformed;

[b] Values represent standardised difference (in SD unit of the metabolic marker) per 1 SD of each saturated fatty acid (SFA). The confidence intervals were corrected for multiple testing based on a familywise error rate of 5%. * $P < 0.05$, ** $P < 0.01$,*** $P < 0.001$.

Model 1, unadjusted;

Model 2, adjusted for age, sex and BMI;

Model 3, model 2 plus physical activity, smoking status, alcohol drinking, family history of diseases and total energy intake;

FBG fasting blood glucose; FINS fasting insulins; HOMA-IR Homeostatic Model Assessment for Insulin Resistance; TG triglycerides; TC total cholesterol; HDL-C high-density lipoprotein cholesterol; LDL-C low-density lipoprotein cholesterol; SFA saturated fatty acid.

Table S3 Association of erythrocyte membrane even-chain saturated fatty acids (per 1 SD difference) with metabolic markers

	Model	C14:0	C16:0	C18:0	Even-chain SFA
		Standardised Difference (CI) [b]	Standardised Difference (CI) [b]	Standardised Difference (CI) [b]	Standardised Difference (CI) [b]
FBG (mmol/L)[a]	Model 1	0.092(0.023,0.162)	-0.028(-0.098,0.041)	-0.071(-0.140,-0.001)	-0.059(-0.128,0.010)
	Model 2	0.066(-0.002,0.134)	-0.016(-0.083,0.052)	-0.071(-0.138,-0.004)	-0.050(-0.117,0.017)
	Model 3	0.049(-0.019,0.116)	-0.015(-0.082,0.051)	-0.057(-0.124,0.010)	-0.042(-0.109,0.024)
FINS (μU/ml)[a]	Model 1	0.154(0.085,0.222)	0.024(-0.045,0.094)	0.033(-0.036,0.103)	0.044(-0.026,0.114)
	Model 2	0.135(0.068,0.202)	0.033(-0.034,0.100)	0.020(-0.047,0.087)	0.043(-0.024,0.110)
	Model 3	0.134(0.066,0.201)***	0.031(-0.036,0.098)	0.033(-0.035,0.100)	0.048(-0.019,0.115)
HOMA-IR[a]	Model 1	0.166(0.097,0.234)	0.012(-0.057,0.082)	0.007(-0.063,0.076)	0.020(-0.050,0.089)
	Model 2	0.141(0.075,0.207)	0.024(-0.042,0.090)	-0.005(-0.072,0.061)	0.022(-0.044,0.088)
	Model 3	0.134(0.068,0.200)***	0.022(-0.043,0.088)	0.010(-0.056,0.077)	0.029(-0.037,0.095)
TG (mmol/L)[a]	Model 1	0.428(0.365,0.490)	0.074(0.005,0.143)	-0.184(-0.253,-0.116)	-0.027(-0.096,0.043)
	Model 2	0.429(0.368,0.489)	0.082(0.016,0.149)	-0.182(-0.248,-0.116)	-0.018(-0.085,0.049)
	Model 3	0.416(0.355,0.476)***	0.082(0.016,0.147)*	-0.167(-0.233,-0.102)***	-0.010(-0.076,0.056)

TC (mmol/L)	Model	1	0.202(0.134,0.270)	0.015(-0.055,0.084)	-0.043(-0.113,0.026)	-0.005(-0.074,0.065)
	Model	2	0.186(0.118,0.254)	0.021(-0.048,0.090)	-0.040(-0.109,0.029)	0.002(-0.067,0.070)
	Model	3	0.184(0.115,0.252)***	0.019(-0.050,0.087)	-0.033(-0.102,0.037)	0.004(-0.065,0.072)
HDL-C (mmol/L)	Model	1	0.011(-0.058,0.081)	0.012(-0.057,0.082)	0.037(-0.032,0.107)	0.031(-0.038,0.101)
	Model	2	0.002(-0.060,0.064)	0.007(-0.054,0.068)	0.034(-0.027,0.096)	0.025(-0.036,0.086)
	Model	3	0.009(-0.053,0.071)	0.007(-0.054,0.068)	0.024(-0.037,0.086)	0.019(-0.042,0.080)
LDL-C (mmol/L)	Model	1	0.119(0.050,0.188)	0.008(-0.062,0.077)	0.002(-0.067,0.072)	0.012(-0.057,0.082)
	Model	2	0.101(0.032,0.171)	0.016(-0.053,0.085)	0.008(-0.061,0.076)	0.021(-0.048,0.090)
	Model	3	0.099(0.029,0.168)**	0.013(-0.056,0.082)	0.017(-0.052,0.086)	0.024(-0.045,0.092)

[a]FBG,FINS,HOMA-IR,TG were log-transformed;

[b] Values represent standardised difference (in SD unit of the metabolic marker) per 1 SD of each saturated fatty acid (SFA). The confidence intervals were corrected for multiple testing based on a familywise error rate of 5%. * $P < 0.05$, ** $P < 0.01$,*** $P < 0.001$.

Model 1, unadjusted;

Model 2, adjusted for age, sex and BMI;

Model 3, model 2 plus physical activity, smoking status, alcohol drinking, family history of diseases and total energy intake;

FBG fasting blood glucose; FINS fasting insulins; HOMA-IR Homeostatic Model Assessment for Insulin Resistance; TG triglycerides; TC total cholesterol; HDL-C high-density lipoprotein cholesterol; LDL-C low-density lipoprotein cholesterol; SFA saturated fatty acid.

Table S4 Association of erythrocyte membrane very-long-chain saturated fatty acids with (per 1 SD difference) with metabolic markers

	Model		C20:0<br>Standardised Difference (CI) [b]	C22:0<br>Standardised Difference (CI) [b]	C23:0<br>Standardised Difference (CI) [b]	C24:0<br>Standardised Difference (CI) [b]	Very-long-chain SFA<br>Standardised Difference (CI) [b]
FBG (mmol/L)[a]	Model	1	-0.108(-0.177,-0.038)	-0.009(-0.078,0.061)	-0.035(-0.104,0.035)	0.102(0.033,0.172)	0.067(-0.002,0.137)
	Model	2	-0.098(-0.166,-0.029)	-0.015(-0.084,0.053)	-0.025(-0.093,0.044)	0.070(0.002,0.138)	0.042(-0.026,0.110)
	Model	3	-0.101(-0.168,-0.033)**	-0.038(-0.106,0.030)	-0.031(-0.099,0.036)	0.047(-0.020,0.114)	0.018(-0.050,0.085)

FINS (μU/ml)[a]	Model 1	-0.006(-0.075,0.064)	0.031(-0.039,0.100)	0.109(0.040,0.179)	0.104(0.035,0.174)	0.093(0.024,0.162)
	Model 2	0.005(-0.063,0.073)	0.032(-0.036,0.100)	0.100(0.033,0.168)	0.080(0.013,0.147)	0.075(0.008,0.142)
	Model 3	0.011(-0.057,0.079)	0.028(-0.041,0.096)	0.105(0.038,0.173)**	0.075(0.008,0.143)*	0.071(0.003,0.138)*
HOMA-IR[a]	Model 1	-0.040(-0.109,0.030)	0.024(-0.045,0.094)	0.086(0.016,0.155)	0.126(0.056,0.195)	0.104(0.035,0.173)
	Model 2	-0.027(-0.094,0.040)	0.023(-0.044,0.091)	0.081(0.014,0.148)	0.094(0.027,0.160)	0.080(0.013,0.146)
	Model 3	-0.023(-0.090,0.044)	0.012(-0.055,0.080)	0.083(0.016,0.150)*	0.082(0.016,0.148)*	0.068(0.002,0.135)*
TG (mmol/L)[a]	Model 1	-0.201(-0.270,-0.133)	-0.142(-0.211,-0.074)	-0.076(-0.145,-0.006)	0.050(-0.019,0.120)	-0.018(-0.087,0.052)
	Model 2	-0.170(-0.237,-0.103)	-0.124(-0.192,-0.056)	-0.050(-0.118,0.018)	0.024(-0.044,0.091)	-0.029(-0.096,0.039)
	Model 3	-0.170(-0.236,-0.103)***	-0.131(-0.198,-0.063)***	-0.042(-0.110,0.025)	0.010(-0.057,0.077)	-0.041(-0.108,0.026)
TC (mmol/L)	Model 1	-0.037(-0.106,0.033)	0.101(0.032,0.171)	-0.043(-0.112,0.027)	0.122(0.053,0.191)	0.117(0.048,0.186)
	Model 2	-0.059(-0.128,0.011)	0.075(0.005,0.145)	-0.046(-0.116,0.023)	0.112(0.043,0.181)	0.100(0.032,0.169)
	Model 3	-0.052(-0.122,0.018)	0.074(0.003,0.144)*	-0.041(-0.111,0.029)	0.107(0.038,0.177)**	0.097(0.028,0.167)**
HDL-C (mmol/L)	Model 1	0.129(0.060,0.198)	0.086(0.017,0.155)	0.033(-0.036,0.103)	-0.058(-0.128,0.011)	-0.011(-0.081,0.059)
	Model 2	0.053(-0.009,0.115)	0.02(-0.043,0.082)	-0.012(-0.074,0.050)	-0.032(-0.093,0.030)	-0.016(-0.077,0.046)
	Model 3	0.054(-0.008,0.116)	0.023(-0.039,0.086)	-0.016(-0.078,0.046)	-0.027(-0.089,0.035)	-0.011(-0.073,0.051)
LDL-C (mmol/L)	Model 1	-0.039(-0.108,0.031)	0.119(0.050,0.188)	-0.036(-0.106,0.033)	0.172(0.104,0.241)	0.161(0.093,0.230)
	Model 2	-0.044(-0.113,0.026)	0.107(0.038,0.177)	-0.028(-0.098,0.041)	0.157(0.088,0.225)	0.145(0.077,0.214)
	Model 3	-0.036(-0.106,0.035)	0.107(0.037,0.177)**	-0.022(-0.092,0.048)	0.153(0.084,0.222)***	0.144(0.075,0.213)***

[a]FBG,FINS,HOMA-IR,TG were log-transformed;

[b] Values represent standardised difference (in SD unit of the metabolic marker) per 1 SD of each saturated fatty acid (SFA). The confidence intervals were corrected for multiple testing based on a familywise error rate of 5%. * $P < 0.05$, ** $P < 0.01$,*** $P < 0.001$.

Model 1, unadjusted;

Model 2, adjusted for age, sex and BMI;

Model 3, model 2 plus physical activity, smoking status, alcohol drinking, family history of diseases and total energy intake;

FBG fasting blood glucose; FINS fasting insulins; HOMA-IR Homeostatic Model Assessment for Insulin Resistance; TG triglycerides; TC total cholesterol; HDL-C high-density lipoprotein cholesterol; LDL-C low-density lipoprotein cholesterol; SFA saturated fatty acid.

Table S5 Sensitivity analysis for the association of erythrocyte membrane saturated fatty acid groups and metabolic markers

		Odd-chain SFA	Even-chain SFA	Very-long-chain SFA
Marker	Model	Standardised Difference (CI)[b]	Standardised Difference (CI)[b]	Standardised Difference (CI)[b]
FBG(mmol/L)[a]	Model 3	-0.099(-0.169,-0.030)	-0.042(-0.109,0.024)	0.018(-0.050,0.085)
	Model 3a	-0.095(-0.167,-0.023)	-0.039(-0.106,0.027)	0.028(-0.040,0.096)
	Model 3b	-0.093(-0.163,-0.024)	-0.040(-0.107,0.026)	0.027(-0.041,0.094)
	Model 3c	-0.106(-0.175,-0.036)	-0.040(-0.106,0.026)	0.014(-0.053,0.082)
	Model 3d	-0.098(-0.168,-0.029)	-0.042(-0.108,0.024)	0.017(-0.050,0.084)
	Model 3e	-0.113(-0.183,-0.043)	-0.041(-0.108,0.026)	0.023(-0.045,0.091)
	Model 3f	-0.102(-0.100,-0.031)	-0.034(-0.100,0.033)	0.033(-0.035,0.101)
FINS(mmol/L)[a]	Model 3	-0.042(-0.112,0.027)	0.048(-0.019,0.115)	0.071(0.003,0.138)
	Model 3a	-0.046(-0.119,0.027)	0.048(-0.019,0.115)	0.070(0.001,0.138)
	Model 3b	-0.046(-0.117,0.024)	0.048(-0.019,0.114)	0.069(0.001,0.137)
	Model 3c	-0.044(-0.114,0.027)	0.048(-0.018,0.115)	0.070(0.003,0.138)
	Model 3d	-0.037(-0.107,0.033)	0.048(-0.018,0.115)	0.069(0.002,0.137)
	Model 3e	-0.057(-0.128,0.014)	0.050(-0.018,0.118)	0.077(0.009,0.146)
	Model 3f	-0.061(-0.131,0.010)	0.054(-0.013,0.121)	0.081(0.012,0.149)
HOMA-IR	Model 3	-0.070(-0.138,-0.001)	0.029(-0.037,0.095)	0.068(0.002,0.135)
	Model 3a	-0.071(-0.143,0.000)	0.030(-0.036,0.096)	0.071(0.003,0.138)
	Model 3b	-0.071(-0.140,-0.002)	0.029(-0.037,0.095)	0.069(0.002,0.137)
	Model 3c	-0.073(-0.142,-0.004)	0.030(-0.036,0.096)	0.067(0.000,0.134)
	Model 3d	-0.065(-0.134,0.004)	0.029(-0.036,0.095)	0.067(0.000,0.133)
	Model 3e	-0.087(-0.157,-0.016)	0.031(-0.037,0.098)	0.076(0.008,0.144)
	Model 3f	-0.087(-0.156,-0.017)	0.037(-0.029,0.102)	0.082(0.015,0.149)
TG(mmol/L)[a]	Model 3	-0.115(-0.184,-0.046)	-0.010(-0.076,0.056)	-0.041(-0.108,0.026)
	Model 3a	-0.102(-0.174,-0.031)	-0.011(-0.077,0.055)	-0.045(-0.112,0.023)

	Model 3b	-0.123(-0.192,-0.053)	-0.012(-0.078,0.054)	-0.050(-0.117,0.018)
	Model 3c	-0.132(-0.200,-0.065)	-0.003(-0.068,0.062)	-0.052(-0.118,0.014)
	Model 3d	-0.105(-0.174,-0.036)	-0.010(-0.076,0.056)	-0.042(-0.109,0.025)
	Model 3e	-0.114(-0.183,-0.045)	-0.009(-0.076,0.057)	-0.038(-0.105,0.029)
	Model 3f	-0.111(-0.181,-0.041)	-0.002(-0.068,0.064)	-0.024(-0.091,0.044)
TC(mmol/L)	Model 3	-0.029(-0.101,0.043)	0.004(-0.065,0.072)	0.097(0.028,0.167)
	Model 3a	-0.043(-0.118,0.032)	0.004(-0.065,0.073)	0.092(0.021,0.162)
	Model 3b	-0.037(-0.109,0.036)	0.003(-0.066,0.072)	0.092(0.022,0.162)
	Model 3c	-0.052(-0.122,0.019)	0.012(-0.055,0.080)	0.083(0.015,0.152)
	Model 3d	-0.034(-0.107,0.038)	0.004(-0.065,0.073)	0.097(0.027,0.166)
	Model 3e	-0.032(-0.104,0.040)	0.004(-0.065,0.072)	0.096(0.027,0.165)
	Model 3f	-0.047(-0.120,0.026)	0.009(-0.060,0.077)	0.105(0.034,0.175)
HDL-C(mmol/L)	Model 3	0.057(-0.007,0.121)	0.019(-0.042,0.080)	-0.011(-0.073,0.051)
	Model 3a	0.036(-0.031,0.102)	0.017(-0.044,0.078)	-0.017(-0.079,0.046)
	Model 3b	0.055(-0.009,0.119)	0.019(-0.042,0.080)	-0.014(-0.077,0.048)
	Model 3c	0.059(-0.005,0.123)	0.018(-0.043,0.079)	-0.010(-0.072,0.052)
	Model 3d	0.045(-0.019,0.109)	0.019(-0.042,0.079)	-0.009(-0.071,0.052)
	Model 3e	0.064(-0.001,0.129)	0.017(-0.045,0.079)	-0.017(-0.080,0.046)
	Model 3f	0.059(-0.006,0.124)	0.014(-0.047,0.075)	-0.020(-0.083,0.042)
LDL-C(mmol/L)	Model 3	-0.049(-0.121,0.024)	0.024(-0.045,0.092)	0.144(0.075,0.213)
	Model 3a	-0.054(-0.129,0.021)	0.023(-0.046,0.092)	0.144(0.074,0.214)
	Model 3b	-0.054(-0.127,0.018)	0.023(-0.046,0.092)	0.142(0.072,0.212)
	Model 3c	-0.068(-0.139,0.003)	0.031(-0.037,0.099)	0.132(0.064,0.201)
	Model 3d	-0.054(-0.127,0.018)	0.024(-0.045,0.092)	0.143(0.074,0.213)
	Model 3e	-0.058(-0.130,0.014)	0.024(-0.045,0.093)	0.145(0.076,0.214)
	Model 3f	-0.077(-0.150,-0.005)	0.032(-0.037,0.100)	0.156(0.086,0.226)

[a]FBG, FINS, HOMA-IR, TG were log-transformed;

[b] Values represent standardised difference (in SD unit of the metabolic marker) per 1 SD of each saturated fatty acid (SFA) group: odd-chain SFA (C15:0 + C17:0), even-chain SFA (C14:0 + C16:0 + C18:0), very-long-chain SFA (C20:0 + C22:0 + C23:0 + C24:0). The confidence intervals were corrected for multiple testing based on a familywise error rate of 5%.

Sensitivity analyses were conducted based on model 3.

Model 3, adjusted for age, sex, body-mass index, physical activity, smoking status, alcohol drinking, family history of diseases and total energy intake;

Model 3a, model 3 plus a variety of dietary factors, including dairy intake, red and processed meat intake, fruit and vegetable intake, vegetable oil intake;

Model 3b, model 3 plus dietary fiber as a covariate;

Model 3c, model 3 plus self-reported hyperlipidaemia as a covariate;

Model 3d, model 3 plus central obesity as a covariate;

Model 3e, model 3 replacing BMI with central obesity ;

Model 3f, model 3 plus mutual adjustment for the other two saturated fatty acid groups;

FBG fasting blood glucose; FINS fasting insulins; HOMA-IR Homeostatic Model Assessment for Insulin Resistance; TG triglycerides; TC total cholesterol; HDL-C high-density lipoprotein cholesterol; LDL-C low-density lipoprotein cholesterol; SFA saturated fatty acid.

Figure S1 Association of erythrocyte membrane saturated fatty acid groups with metabolic markers by subgroups of alcohol intake and sex

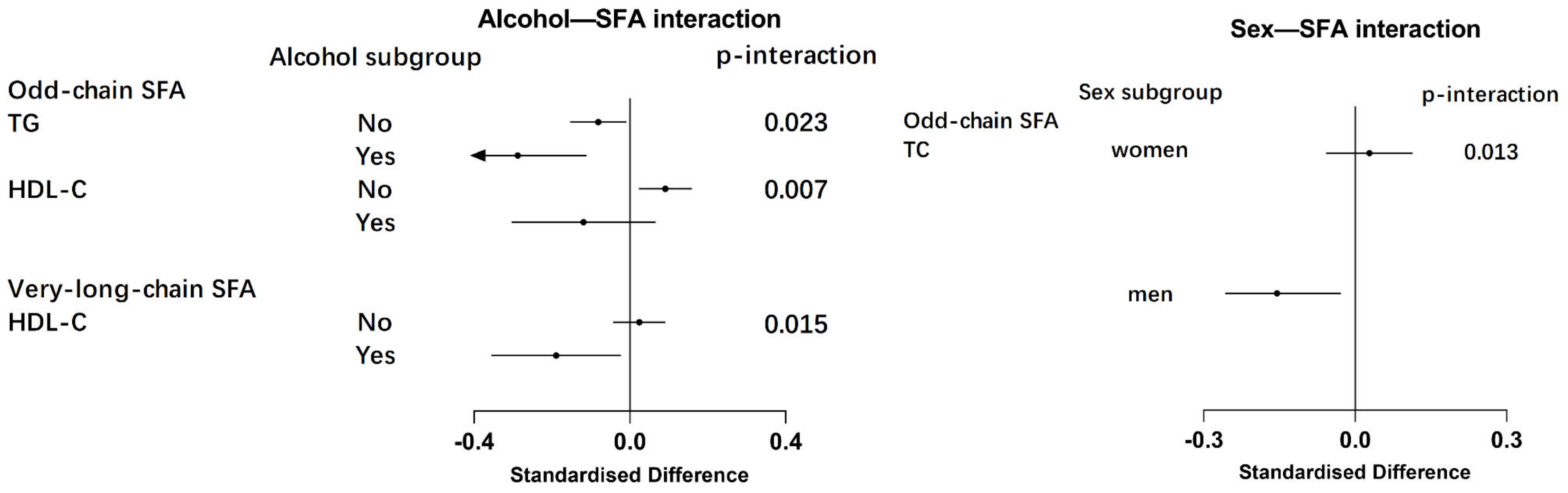

Standardised difference is the difference (in SD unit of the metabolic marker) per 1 SD of the saturated fatty acid (SFA) group: odd-chain SFA (C15:0 + C17:0), very-long-chain SFA (C20:0 + C22:0 + C23:0 + C24:0). Based on the significant interaction observed ($p<0.05$), we presented standardized difference of the metabolic marker per 1 SD of fatty acid by alcohol intake and sex subgroups. TG was log-transformed. TG, triglycerides; TC, total cholesterol; HDL-C, high-density lipoprotein cholesterol; LDL-C, low-density lipoprotein cholesterol;
